# Insanity and Temperament. II.—The Artistic Temperament


**Published:** 1916-05-13

**Authors:** 


### INSANITY AND TEMPERAMENT.

### II. The Artistic Temperament.*

It would be a great mistake to suppose that an artist necessarily possesses the artistic temperament, or that the possessor of this temperament is necessarily an artist. Many artists are very respectable and worthy persons, as excellent in morals as they are in ability; and the man of artistic temperament is usually neither respectable nor worthy, and is never excellent in morals, though he has often a fair share—sometimes a considerable share—of ability. The man of artistic temperament has, in common with the artist, his sensitiveness to sensuous impressions, his appreciation of beauty and grace, of sound, colour, and form. He is a potential artist, and usually is more or less an actual artist in an amateur way; but he has not the industry or application to become an artist in the full sense. He may, and usually does, produce verses, essays, drawings, pictures, music, or other artistic products; but whatever he produces is stamped with the ineffaceable mark of the amateur. It is important not to confuse the artistic temperament with the temperament of the artist, which is a very different thing, and a much greater thing.

### The Feline Character.

If every man embodies the traits of some animal, and will at his death be reincarnated as the animal whose nature he shares, then the possessor of the artistic temperament will undoubtedly spend the next phase of his existence as a cat. He has the cat's self-absorption and aloofness from his fellows; the cat's indifference to social ties and obligations; the cat's sleekness and sedulous care of its person; the cat's incapacity for steady industry, and habit of acting by fits and starts; the cat's luxuriousness and self-indulgence; the cat's fondness for play; and usually the cat's dishonesty and cruelty.

The man of artistic temperament has usually been a spoilt child, and no doubt a good deal of the undisciplined excesses of his adult life are due to the want of discipline in his childhood; but his traits are inborn, and though judicious discipline in childhood might subdue them, no discipline would eradicate them. Very often in his childhood he has been puny: his health has been delicate, and so he has been indulged, has escaped the wholesome discipline of school, and has been excused from many a punishment that a healthy child would have suffered. Often he is the only son, and has been indulged on this account: often he has had a doting mother: often he has lost his father in early life; but though these conditions all favour the development of the artistic temperament, they do not create it. The disposition is inborn, and though it may be minimised by a sound and judicious bringing up, and fostered by an indulgent and foolish education, there is no reason to suppose that it can be either created or eradicated.

The artistic temperament is certainly not strongly hereditary, and it may be doubted whether it is hereditary at all. It may appear sporadically in one member of a large family, the rest of whom are normal; it may appear in children in whose parents it is absent, and may be absent in the children of a father who possesses it in high degree. It is more frequent in men than in women; but, whatever its origin, it is a calamity to the family of its possessor, though it is not necessarily a calamity to the possessor himself. It does often bring him to irretrievable disaster, it is true, but it does not necessarily do so; and the very selfishness which is an integral and conspicuous element in it may, if combined with a moderate share of prudence, secure for its possessor that ease and comfort for which he most craves, and make his life, if not prosperous or successful, at least enjoyable. He does not always wreck his own life, but if the temperament is highly developed he will infallibly wreck the lives of others.

### The Indulgence of Self.

For the keynote of the artistic temperament is selfishness, the dominant is self-indulgence, and the sub-dominant sensitiveness to sensuous impressions. If men are divided into those who feel, those who think, and those who act, then the men of this temperament belong to the first class. They are, indeed, actors, but they are not men of action. They are actors in the histrionic sense. They pose and grimace. They constantly seek to attract attention and interest from others; but they are not men of action. They are saunterers. They are lookers-on at the battle of life, and restrict their exertions to criticising and sneering at those who do the fighting. In as far as they act at all, their action is recreative. They are dexterous and nimble, and can do many useless things neatly and well. They are skilful at games, and so they should be, for they spend on games a very disproportionate share of their time. They have the musical faculty, and can sing and play some musical instrument, perhaps several musical instruments, with easy dexterity. They draw and paint, write verses and plays and novels, may even be witty and brilliant in conversation; but whatever they do, they do as amateurs. They are often very good amateurs, but they are no more than amateurs even in the profession they may adopt. To attain professional rank, even in an artistic or a purely recreative avocation, such as that of the conjurer, requires persistence in steady industry; and of steady industry they are incapable. They will occupy themselves only as long as the occupation is pleasant and congenial to them. The moment it begins to pall and become tedious, it is thrown aside. This is not work: it is occupation, but it is not work, for work is doing that which is distasteful. If they work at all, it is by fits and starts, in snatches of brief duration, and in the doing they are inefficient, inattentive, procrastinating, dilatory, and leave their tasks half done. As long as the work has the attraction of

* The first article of this series appeared on May 6.

novelty, they will pursue it with enthusiasm and energy, but they do not persist. As soon as it becomes tedious, it is abandoned. They are unmethodical. They have neither the foresight to devise a plan for themselves nor the tenacity to adhere to a plan made for them, and are too lazy to be orderly in business.

In Business.

In business affairs they are unthrifty and extravagant. They spend disproportionately on present indulgence, on personal adornment and pleasure, and have little regard to future wants, and none whatever for the welfare of others, even of those nearest to them. They borrow without any intention to repay: they cadge without shame, and care not of how much they may deprive others, so that their own immediate wants are satisfied. Anyone—father, mother, sister, wife, or even child—may work for them or want for them, and they take all that is given to them as their natural right, for which they express no gratitude, for they feel none; and for which they neither own nor feel obligation. In the extreme instances of this temperament, even the ordinary obligations of morality are not acknowledged as binding or applicable to themselves, though they are quick to resent any relaxation of these rules by which they may suffer. Such persons will rob, and forge, and swindle without any acknowledgment, without, it seems, any realisation, that they are doing wrong, or that they are doing anything that their victims have any right or reason to resent; nor do they express or feel any gratitude to the relatives who rescue them from the legal consequences of their depredations. So far from being grateful for what is done for them, they are resentful and indignant that more was not done.

Insensitive to Honour.

As none of them is sensitive to the demands of honour, and some are indifferent to the obligations of honesty, it will easily be believed that they are no devotees to truth. They are, indeed, facile, plausible, and unblushing liars, and display little shame or embarrassment when their lies are exposed. They seem not to appreciate the difference between truth and falsehood. They lie and brag about their own achievements, for they are always conceited; and they lie in disparagement of others, for they are always envious of those who are more successful or more esteemed than themselves. The same selfishness and laziness that underlie their action in important matters secure that they are wanting in manners; for good manners and courtesy mean unselfishness and willingness to take trouble in little things. To a new acquaintance, indeed, especially if they have anything to gain from him, they can for a time be fascinating, for they are ready of tongue, and their sensitiveness to impressions endows them with tact; but as in other things, so in this, sustained effort soon becomes wearisome, and they cannot keep it up. To the feelings of those whom they have no motive to propitiate they are frankly indifferent, and care nothing whether they merely jar upon the taste, or whether they outrage the deep consciousness of right and wrong.

The character that is here described as that of the artistic temperament is not an attractive one, and perhaps may be considered to be painted in colours too gloomy; but there are few who cannot reckon among their acquaintances one or more to whom the description applies. The possessor of this temperament has few redeeming features to set off against his defects, nor are his good qualities very important. For a time, and until his egotism asserts itself, he can be very entertaining and charming. He is a ready talker, sometimes a witty talker. He can talk entertainingly—chiefly about himself, it is true—but he can talk entertainingly, can organise games and amusements and take the lead in them; but here, as a rule, his accomplishments end. He is an acquisition at a dinner party or a picnic, but in any of the serious affairs of life he is a clog and a hindrance, if no worse. He is usually clever, but he is always incapable, and this leads us to the very important distinction between cleverness and capability, which will occupy a subsequent article.

